# Long term consistency and location specificity of equine gluteus medius muscle activity during locomotion on the treadmill

**DOI:** 10.1186/s12917-018-1443-y

**Published:** 2018-04-06

**Authors:** Rebeka R. Zsoldos, Anna Voegele, Bjoern Krueger, Ulrike Schroeder, Andreas Weber, Theresia F. Licka

**Affiliations:** 1 Department of Sustainable Agricultural Systems, Section Livestock Sciences, University of Natural Resources and Life Sciences Vienna, Vienna, Austria; 2 Multimedia, Simulation and Virtual Reality Group, Institute of Computer Science II, University of Bonn, Bonn, Germany; 3 Gokhale Method Institute, Stanford, CA USA; 4 Department for Companion Animals and Horses, University of Veterinary Medicine Vienna, Vienna, Austria; 5 Royal (Dick) School of Veterinary Studies, The University of Edinburgh, Edinburgh, Scotland UK

**Keywords:** Surface electromyography, Horse, Gluteus medius, Walk, Trot, Locomotion, Scar, Gaussian distribution

## Abstract

**Background:**

The equine m. gluteus medius (GM) is the largest muscle of the horse, its main movement function is the extension of the hip joint. The objective of the present study was to measure equine GM activity in three adjacent locations on GM during walk and trot on a treadmill, in order to document potential differences. Fourteen Haflinger mares were measured using surface electromyography and kinematic markers to identify the motion cycles on three occasions over 16 weeks. The electrodes were placed on left and right gluteus medius muscle over the middle of its widest part and 5 cm lateral and medial of it. For data processing, electrical activity was normalised to its maximum value and timing was normalised to the motion cycle. A Gaussian distribution approach was used to determine up to 10 modes of focussed activity, and results were analysed separately for stance and swing phase of the ipsilateral hindlimb.

**Results:**

Fair reliability was found for mean mode values (Cronbach’s alpha = 0.66) and good reliability was found for mean mode locations (Cronbach’s alpha = 0.71) over the three data collection days. The magnitude of muscle activity identified as mean mode value was much larger at trot than at walk, and mean mode value was significantly different between stance phases of walk and trot for all electrode positions (*p* < 0.01). The pattern of muscle activity identified as mean mode location was significantly different for walk and trot at all electrode positions, both during stance and swing phases (*p* < 0.001). This indicates the different timing pattern between the gaits. Results of the three electrode positions on the same muscle during each gait were not significantly different when comparing the same measurement.

**Conclusions:**

The middle of the equine GM does not show any indication of functional differentiation during walk and trot on a treadmill; this might be due to lack of segmentation as such, or due to lack of need for segmented use for these very basic main tasks of the muscle. The reliability of the sEMG measurements over several weeks was fair to good, an indication for the robustness of the methodology.

## Background

The equine m. gluteus medius (GM) is the largest muscle of the horse, its main movement function is the extension of the hip joint. In the unloaded limb GM contraction swings the limb towards caudal for e.g. kicking out and in the weight bearing limb it pushes the body mass forward during locomotion. Besides that, this muscle also creates some abduction of the limb. As with all leg muscles, it has a function in stabilising the limb during the stance phase of locomotion, during dynamic movements such as the capriole (i.e. the horse leaping from the ground and kicking out with its hind legs) and during static postures such as the levade (i.e. the horse lifting its forelegs from the ground and balancing on its deeply bent hindlimbs).

The equine GM creates the visible contour of the equine croup, even though it is partly covered by the thin m. gluteus superficialis and by the gluteal fascia (the caudal extension of the thoracolumbar fascia) [1]. It is a well and frequently used candidate for surface electromyography (sEMG), and its electrical activity has been measured in a large number of studies. Furthermore, the importance of this muscle has been documented in several studies: Payne et al. [1] estimated the GM has the highest capacity for force production (11,900 N) in the horse due to its very large physiological cross sectional area and long fascicles. Also, lengths and contraction velocities of muscle fibres have a dramatic effect on such muscle force generation [2]. The use of the equine GM during increasing speed and treadmill gradient was documented [3–6], and both increases in speed and treadmill inclination were found to increase EMG activity due to the higher workload of the GM. In the human gluteus maximus, Bartlett et al. [7] also found that increased muscle activity is related to the speed and intensity of the movement, somewhat independent of the gait (walk or run). Recently, integrated EMG values for GM in Thoroughbred racehorses were documented to increase uphill, but not decrease downhill compared to flat work on the treadmill [6]. This may indicated the increased need for limb stabilisation downhill compared to flat or uphill work.

Compartmentalisation, or the division of muscles into anatomically and/or functionally distinct sub-volumes, is an important aspect of organization and control in many muscles [8]; this has been shown e.g. for the human deltoid muscle [9], where muscle fibres converge in a single insertion similar to the gluteus medius. Sub-regions are usually supplied by different “primary” branches of a motor nerve (and often also by different blood vessels). In principle this would allow detailed control of these regions by the central nervous system both during volitional and central pattern generated movements. Most commonly, the term compartmentalisation has been reserved to describe the situation in which different sets of motor units occupy different sub-volumes within a single muscle [10]. The presence of regional differences of muscle activities during locomotion has been shown in the longissimus dorsi muscle [11]. There each section is innervated by one spinal nerve and served by one artery (Ramus dorsalis of the Arteria costoabdominalis dorsalis) serves one section [12, 13]. The presence of such functional subunits has not been documented in the equine GM during locomotion. However, in the GM of two Thoroughbred horses regions with different fibre type compositions were identified, indicative of a degree of specialisation of these regions [14]. This fibre typing was continued in a variety of horse breeds, showing more Type I fibres (ideal for aerobic, slower, continuous work such as joint stabilisation) located deeply and more Type II fibres (better suited to anaerobic, fast bursts of work, such as wide range of motion movements) located superficially in the GM. As this fibre type composition changes with the muscle depth, this will also be the case for the electrical activity, which is influenced by the fibre type [15], however this influence is still under investigation. Besides the metabolic differentiation of fibre types, movement function obviously also depends on the direction of muscle fibres, which varies within the GM due to its different origins with a single insertion point. Different movement functions within a muscle are thought to aid spreading peak muscle load over a larger area as well as over more time to reduce the risk of injury and to improve muscle perfusion.

In very large muscles, such as the equine gluteus medius muscle (GM), regional variations potentially representing functional differences of muscle activities have to be expected. The aim of the present study was therefore to measure the surface muscle activity of the equine GM muscle during walk and trot on the treadmill in three adjacent locations and to document potential differences between these activities. The long term consistency of these findings was investigated by repeating measurements after 8 and 16 weeks.

The following hypotheses were examined: a) there are significant differences in the muscle activities between the three adjacent locations of surface EMG electrodes during locomotion b) the surface muscle activities do not change significantly over 16 weeks within any individual.

## Methods

Horses used for the study were assessed for signs of back pain and/or lameness at walk and trot by two veterinarians (U.S. and T.F.L.) before and after each measurement and found to be without any such signs. Fourteen horses without clinical sign of back pain and lameness were used in this study (Haflinger mares, mean age 8 ± 3 years, confidence interval (CI) (6, 9), range 4–14 years; mean body mass was 463 ± 42 kg, CI (439, 487), range 396–526 kg; mean height at the withers was 131 ± 5 cm, CI (128, 134), range 125–145 cm). Haflinger mares heterozygous positive (polysaccharide storage myopathy type 1, *n* = 7) and negative (control, n = 7) for glycogen synthase (GYS1) mutation were included. As a part of the preceding study using the same data collections [16], the effect of diet was investigated; horses received 6 weeks of carbohydrate rich diet followed by hay only or vice versa, with a wash out period of two weeks between diets. The results of that study have been published [16], and no effect of diet or GYS1 was found; therefore, this factor is not further investigated in the present study. For that study surgical biopsies of GM (removing about 1 cm^3^ of muscle) were taken unilaterally after the second and on the contralateral side after the third data collection, presenting the opportunity to investigate sEMG on one location before and after an intentional surgical muscle lesion. Horses were accustomed to the treadmill and the experimental set up at walk and trot, and prior to the measurements they were warmed up for two to three minutes. For the measurements, horses walked and trotted on the treadmill at their optimum speed [17]. Data collection was carried out on three different data collection days (DCD): first measurement (DCD 1), 8 weeks later the second measurement (DCD2) and 16 weeks after DCD1 the third measurement (DCD3).

### Kinematic measurements

Seven reflective skin markers were positioned on each horse using adhesive tape; one on the forehead, one on the highest point of the withers, on the sacrum were placed and on the lateral side of each hoof to identify motion cycles. Three-dimensional kinematic data at walk (@W) and trot (@T) were collected using ten high-speed cameras recording at 120 Hz. Three-dimensional coordinates of each marker during the time course of each experiment were calculated from the data using kinematic software. These time series were then smoothed by use of a Butterworth low-pass filtered (cut-off frequency, 10 Hz). Based on the vertical movement of the left hind hoof, the motion cycles were cut, and left and right hind stance and swing phases were identified using a custom-made MATLAB script. The duration of each full motion cycle consisting of stance and swing phase was normalised to 100%. In the remainder of the paper, stance phase and swing phase always refer to the ipsilateral hind limb. For each horse and each trial, a minimum of six motion cycles at walk and a minimum of thirteen motion cycles at trot were considered. Per DCD, three consecutive trials were measured in each gait, resulting in a minimum of 18 motion cycles of walk and 39 motion cycles of trot per horse for further analysis.

### Muscle activity measurements

**Fig. 1:**
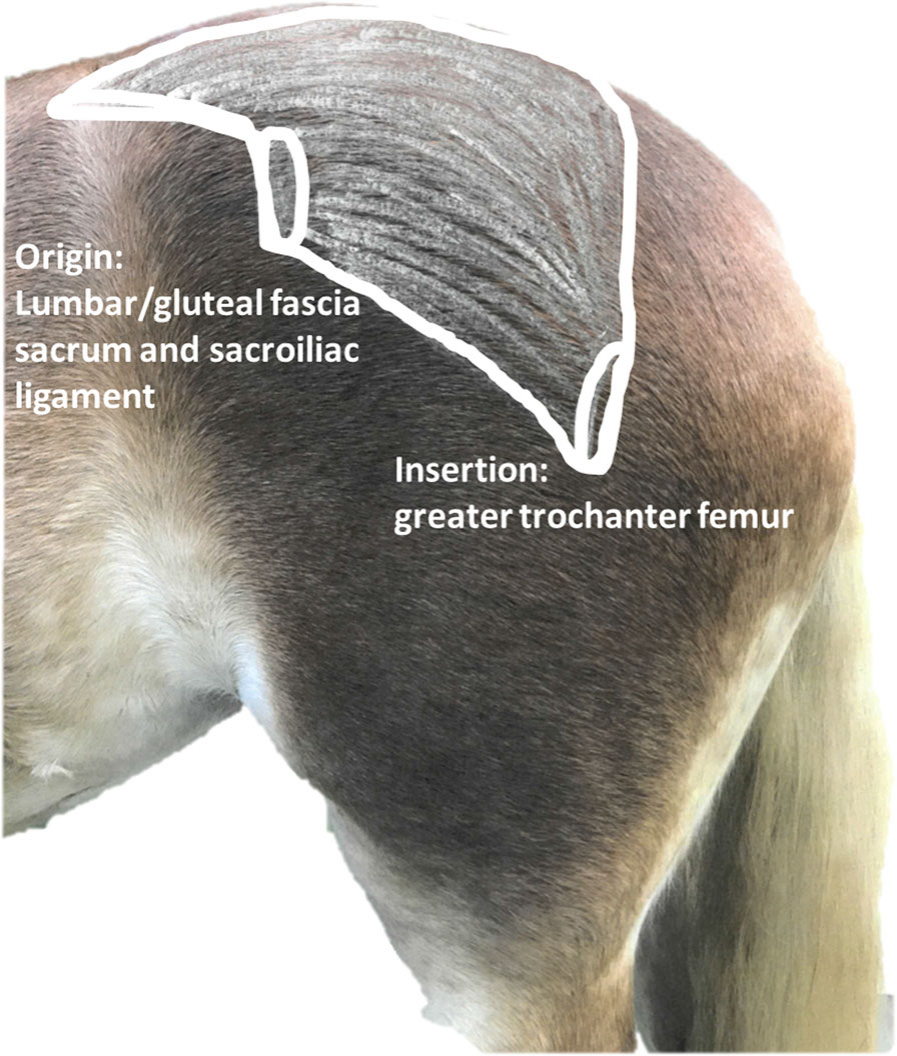
Anatomical representation of the left equine gluteus medius muscle on a Haflinger horse. Origin and insertion of the left equine gluteus medius muscle are highlighted

Before electrode placement hair was removed with electric clippers, the skin was shaved, cleaned with isopropyl alcohol solution with slightly abrasive, roughly woven swabs to reduce the resistance. Surface electromyography activities were collected with wireless electrodes, with each sensor consisting of four parallel silver bars with an integrated amplifier, size 27 × 37 × 15 mm, mass 14.7 g. They were placed parallel to the fibre orientation over the left and right GM. The electrodes were positioned on the skin using the Delsys Adhesive Sensor Interface™ allowing for free skin contact of the silver bars without any solution or electrode gel, as specified by the manufacturer. The exact position of the central electrode was at the midpoint between origin and insertion in the middle of the widest section of the GM; this was identified by palpation of the tuber coxae, tuber sacrale and 3rd trochanter of the femur (Fig. 1).

**Fig. 2:**
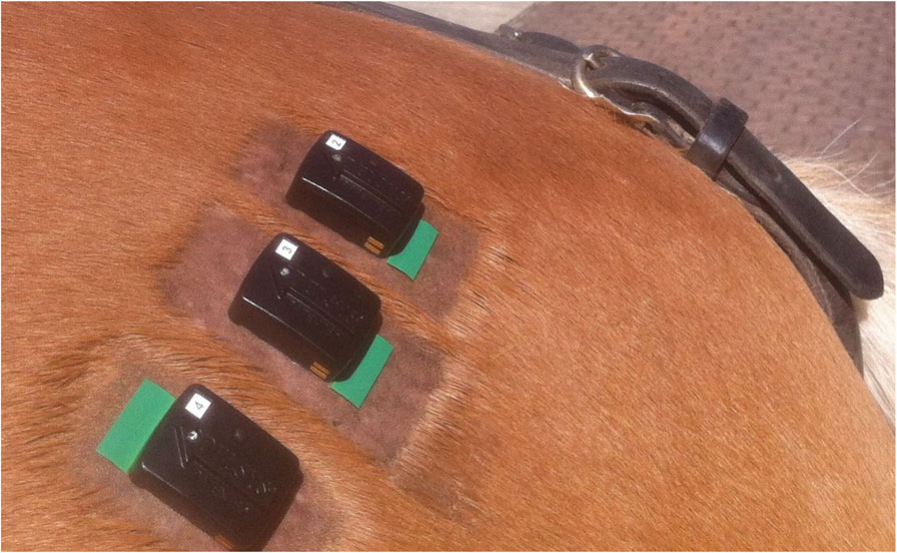
Surface electrodes placed over the left gluteus medius muscle. The three electrodes placed over the left gluteus medius muscle of horse 13 are shown. The sensor number 2 is GM1 (medial to the middle of the muscle), sensor number 3 is GM2 (central on the muscle), and sensor number 4 GM3 (lateral to the middle of the muscle). For each electrode clipped and shaved the rectangles are visible; these were clipped once and remained visible for the rest of data collection days and were only re-shaved. At the last EMG measurement the middle electrode (GM2) was over the eight week old biopsy site

Additional sensors were placed about 5 cm lateral and about 5 cm medial to this central electrode, resulting in three adjacent electrodes termed GM1 (medial on the muscle), GM2 (central on the muscle), and GM3 (lateral on the muscle) (Fig. 2). On DCD3, in all of the horses either the left or the right sided GM2 electrode was positioned over the biopsy scar as described above.

The resulting sEMG signal treated by removal of the direct current offset by subtraction of the mean of the data. Then the sEMG was full-wave rectified and sampling rate was reduced to 120 Hz. A Butterworth low-pass filter was applied (fourth order; cut-off frequency 20 Hz), and the signal was normalised to its unique absolute maximum value at each electrode position for each DCD, resulting in a maximum value of 1 measured at least once per DCD.

The collections of all sEMG cycles of one horse together were used as the basis of Gaussian Mixture Models in the following way: the ten highest sEMG peaks in the set of all sEMG peaks were collected per motion cycle and grouped over the set of all motion cycles per horse, gait and electrode position. This set of peaks was then modelled by a Gaussian mixture distribution [18, 19].

**Fig. 3:**
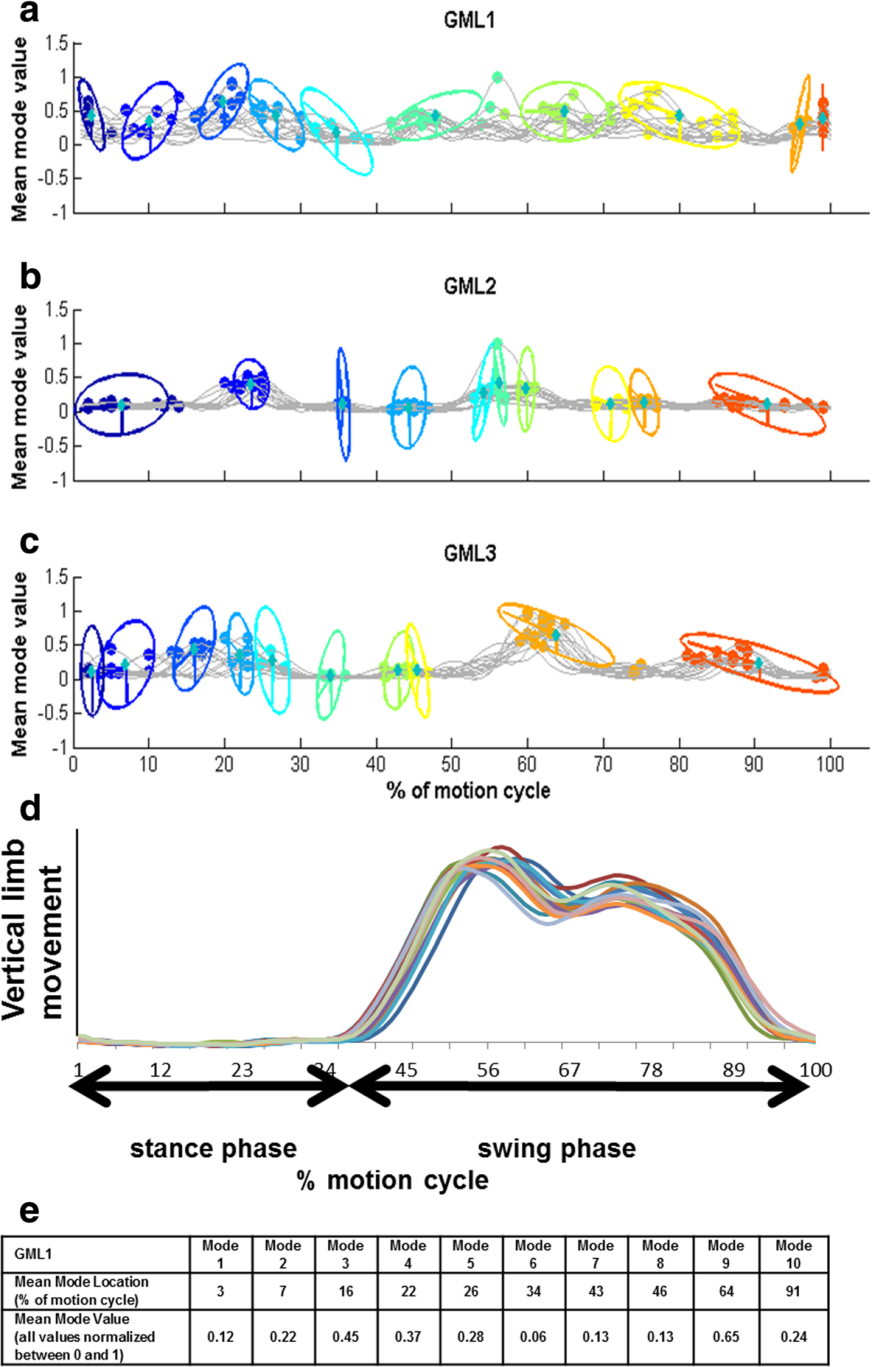
Activity of the left gluteus medius muscle of horse 11. Surface electromyography activity over 15 motion cycles at data collection day 2 trotting on a treadmill is shown; **a**) results of one electrode lateral to the centre of the muscle (GML1), **b**) one over the centre of the muscle (GML2), and **c**) one medial to the centre of the muscle (GML3), **d**) shows the mean vertical movement of the left hindlimb (HHL) during the same measurement and **e**) lists the numerical values for the ten modes evaluated

Results were considered separately for stance phase (St) and swing phase (Sw) of the left (L) and right (R) sides. Mean mode locations (MML), representing the time points within the motion cycle where notable activity is located, were reported in percent of the overall motion cycle duration [18]. Mean mode values (MMV) of the gluteus during the motion cycle are also reported as an arbitrary unit representing the magnitude of the muscle activity (width and height of the trace); the maximum MMV is 1 [18]. Both MML and MMV are illustrated in Fig. 3.

Statistical analyses were done in SPSS. Normality of distributions was tested with Shapiro-Wilk tests. The analysis of parametric data consisted of a T-test for paired samples with Bonferroni corrections (comparisons of walk and trot; comparisons of left and right body side) and repeated measures ANOVA test with Bonferroni corrections (comparisons between the three measurement days). The results of parametric data are presented as mean ± standard deviation (SD). Non-parametric data was tested using a Wilcoxon Signed rank test (comparisons of walk and trot; comparisons of left and right body side) as well as a Friedman’s test (comparisons between the three measurement days). The results of the non-parametric data are presented as mean and interquartile range. In all of the tests above, the level of significance was set at 0.05. Overall reliability of the measurements of MML and MVL on different days was calculated as Cronbach’s alpha coefficients.

## Results

**Fig. 4:**
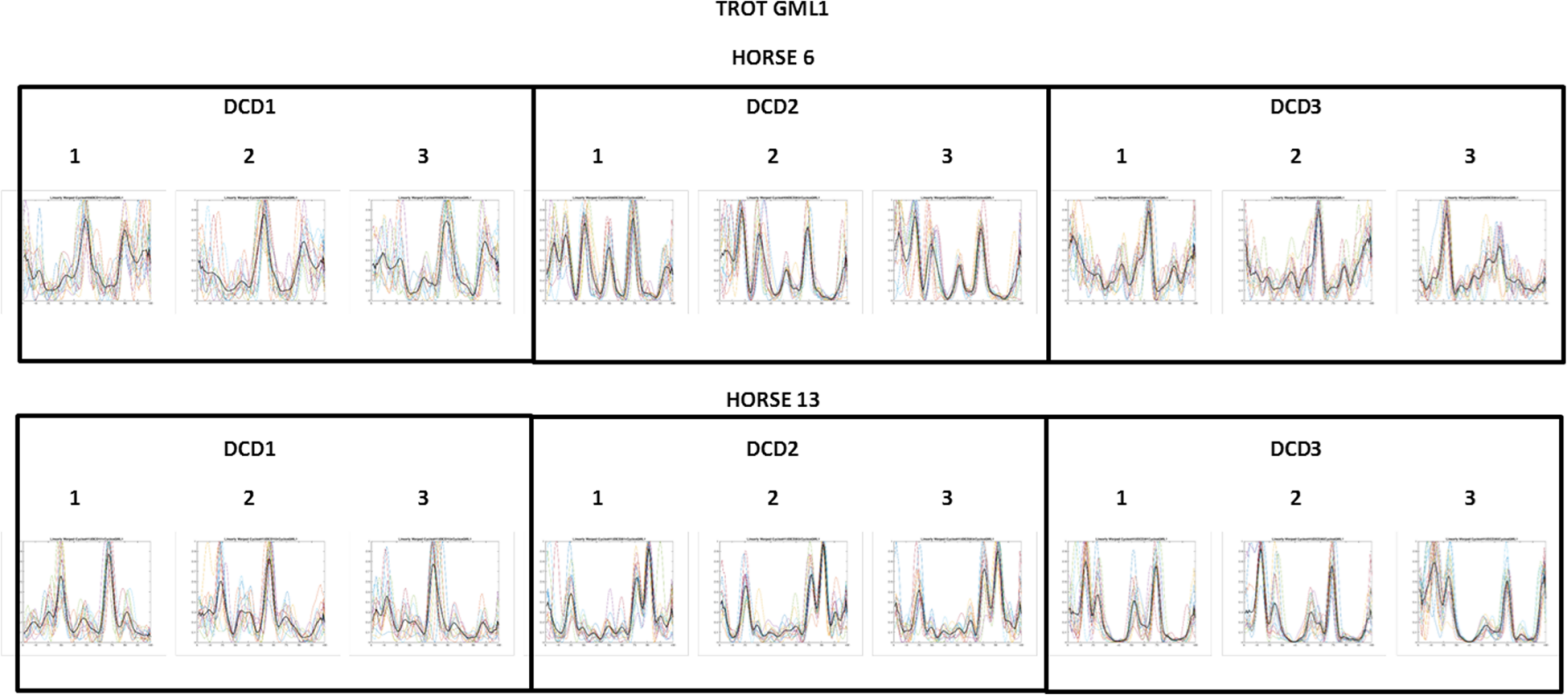
Activity of the left gluteus medius muscle during trot measured three times over 16 weeks. The results obtained from the electrode lateral to the centre of the left gluteus medius muscle (GML1) of horse 6 (top row) and horse 13 (bottom row) are shown for data collection day 1 (DCD1, left three plots), at data collection day 2 (DCD2, middle three plots) and at data collection day 3 (DCD3, right three plots). For each DCD the trot measurements obtained (1, 2, 3) shown, with all motion cycles considered for data analysis. Mean curves of these trials are shown with red on these figures

A minimum of 8 modes were identified on all three locations for all horses in both gaits, with 8 horses showing 10 modes at all locations both at walk and at trot. At walk 4 measurements out of 126 measurements per side showed 9 modes and at trot 1 measurement showed 8 modes and 3 measurements showed 9 modes out of 126, leaving for both gaits 122 measurements with 10 modes. A qualitative difference in the shape of the activity curves at the three electrode positions was clearly visible in all horses (Fig. 4).

### Mean mode value results - magnitude of mode activity

Similar GM activities were found for left and right sides in both gaits, and no significant differences between MMVs of the three electrode positions. Fair reliability was found for MMV over the three DCDs (Cronbach’s alpha = 0.66). Ranges of MMV over all horses over all of the DCDs and all electrode positions were slightly larger at trot than at walk during the stance phase (7%–10% at walk, 8%–18% at trot) but slightly smaller at trot than at walk during the swing phase (11%–16% at walk; 7%–14% at trot).

The muscle activities measured during the stance phase at trot resulted in much larger MMVs compared to walk, and MMV was significantly different between the stance phases of walk and trot for all electrode positions (*p* < 0.01). This illustrates the larger effort of the GM during the stance phase at trot compared to walk. There was no significant difference between the MMVs at walk and trot during the respective swing phase (see Table 1), probably the effect of a faster but more passive forward swing of the hindlimb at trot. Significant differences were found when comparing results of DCD1 and DCD2 with DCD3: DCD1 GM1 MMVSt@T (*p* = 0.001), DCD2 GM1 MMVSt@T (*p* = 0.002), as well as DCD2 GM3 MMVSw@T (*p* = 0.04) (Table 1).

**Table 1:** Mean mode value of the equine gluteus medius muscle activity

Mean mode value		WALK						TROT					
		Stance Phase			Swing Phase			Stance Phase			Swing Phase		
GM1	Mean ± SD	0.29	±	0.09	0.34	±	0.09	0.36	±	0.10	0.33	±	0.07
GM2	Mean ± SD	0.28	±	0.08	0.33	±	0.07	0.35	±	0.09	0.32	±	0.07
GM3	Mean ± SD	0.28	±	0.08	0.34	±	0.09	0.36	±	0.11	0.33	±	0.07

The magnitude of electrical activity (mean mode value) of the equine gluteus medius muscle during swing and stance phase is listed; the maximum value of this arbitrary unit is 1. As there was no statistically significant difference between the values of the left and right side, the average of both sides is given. Mean mode values are shown as (mean ± SD) for electrode lateral to the centre of the muscle (GML1), for electrode over the centre of the muscle (GML2), and for electrode medial to the centre of the muscle (GML3) at walk and trot during the three data collection days

### Mean mode location (MML) results – Pattern of mode activity

Good reliability was found for MML over the three DCDs (Cronbach’s alpha = 0.71). Only four of the 36 DCD comparisons (3 locations, two sides with 6 values for each of the 3 DCDs) were significantly different. All significant differences were found for comparisons with DCD3 results: DCD1 right GM2 MMLSw@W (*p* = 0.01) DCD2 right GM2 MMLSw@W (p = 0.04), both involving results from electrodes over the biopsy site. Even though biopsies had been taken in half of the horses on the left side after DCD 2, no such differences were detected for the left GM2.

Ranges of MML over all horses over all of the DCDs and all electrode positions were slightly larger at walk than at trot and slightly larger during the stance phase (4%–27% at walk; 3%–20% at trot) than during the swing phase (3%–13% at walk; 4%–11% at trot).

Walk and trot resulted in significantly different MMLs at all electrode positions, both during stance and swing phases (*p* < 0.001), indicating the differences in timing pattern. No significant differences between the electrode positions were found.

The time difference between the left and right stance phase and swing phase MMLs was around 50% of the motion cycle at walk and trot (Table 2). This gait dependent shift between left and right hindlimb use at walk and trot was documented as statistically highly significant differences in MML (p < 0.001) in all electrode positions.

**Table 2:** Mean mode location of left and right equine gluteus medius muscle activity per motion cycle

Mean mode location [%]		WALK				TROT			
		Left swing phase	Left stance phase	Right swing phase	Right stance phase	Left swing phase	Left stance phase	Right swing phase	Right stance phase
GM1	Median	76	25	25	76	68	16	16	67
	IQR	3	5	4	4	4	3	5	3
GM2	Median	77	25	26	76	67	16	17	68
	IQR	4	4	3	6	4	5	4	3
GM3	Median	76	25	25	76	68	16	17	67
	IQR	7	3	4	5	3	2	4	3

For walk and trot, the overall mean mode locations in percentage of the total duration of each motion cycle (mean ± SD) results (considering for the three data collection days over 16 weeks with three measurements on each data collection day) obtained from three electrodes (slightly lateral to the centre of the muscle: GM1, over the centre of the muscle: GM2, and slightly medial to the centre of the muscle GM3) are listed for each side

## Discussion

In the present study no difference in GM muscle activities between the left and right sides were found; this indicates symmetrical movements. In an older study on hindlimb lame horses, left and right gluteal muscle activity differed significantly between the lame hindlimb and the sound hindlimb [20], further supporting that bilaterally similar GM activity is associated with absence of lameness.

The results of the present study obtained on a treadmill should not be applied to over ground locomotion without careful consideration. Horses were walking and trotting at their optimum speed on the treadmill [17], and this created a consistent movement pattern with the stance phase standardised to a higher degree than the swing phase. During the stance phase the push-off of the limb is not as strong as it would be during over ground locomotion [21, 22]. Similarly in rats, increased (passive) hip extension at initial limb contact with the treadmill was found when treadmill versus over ground walk was compared [23]. The use of treadmill set-ups for equine locomotion studies has been standard for many years as the laboratory settings with a treadmill allow for exactly repeatable conditions, and the use of measurement equipment is safe and easy in such a laboratory. However, over the last decade, the measurement of horses moving over ground has increased steadily, as the availability of new technologies (such as wireless sensors, and day light kinematic cameras) has expanded. The present study however used a data collection set up optimised for the submaximal exercise tests of the preceding study into the effects of the GYS1 mutation of half of the study horses [16] where the standardisation needed was only achievable on the treadmill. For a study into GM muscle use the selection of a treadmill set up or an over ground set up is relevant, as the function of GM during locomotion is mainly hip extension which is expected to be less forceful on the treadmill compared to over ground.

Also, the degree of GM abduction counteracting the gravitational adduction during the swing phase was expected to be electrode position specific, however this was not found in the present study. The activity level of the muscles was different during the swing and stance phase, and their relative contribution also changed with the gaits. Contrary to earlier assumptions, that the swing phase is mainly passive in any gait, considerable muscle activity during the swing phase was found especially when walking slowly; in humans this active swing phase control at walk was documented [24] and found to require slightly less than one-third of the net energy cost of walking [25]. In guinea fowl, Marsh et al. [26] reported that the swinging limb used an appreciable fraction of the energy in both walking and running. Pontzer [27] showed that the estimated leg-swing energy costs in quadrupeds were lower than in humans where they accounted for 15% (during walking) and 10% (during running) of the cost of locomotion. The results of the present study support this for the equine gluteus activity during walking and trotting on a treadmill: The swing phase MMV was roughly similar at walk and trot while the stance phase MMV was much larger at trot than at walk, creating a relatively smaller contribution of the swing phase muscle activity to the overall energy costs at trot. This is in contrast to a study into the function of the extrinsic hindlimb muscles in trotting dogs, where a small burst of activity of the gluteus superficialis muscle was found within the first 20% of the swing phase [28]. This timing of a relatively small burst of gluteal activity was also found in the gluteus medius activity in the walking and trotting horses in the present study. This may be due to the transfer of function from the gluteus medius muscle to the gluteus superficialis between these two species.

Most studies into sEMG of animals have used data obtained either on a single day of measurements or before and after interventions. Therefore, the reliability of sEMG measurements over 16 weeks (fair for MMV and good for MML) in the present study cannot be directly compared to other studies. However, these results do support the hypothesis of reliability of the EMG signal over many weeks. One study comparing the reliability of sEMG results of human trunk muscles in the same locations twice with a 1-week interval showed excellent reliability for the submaximal MVC while MVC as such had a lower reliability [29] and another study comparing sEMG reliability over two weeks showed high reliability of MVC of trunk muscle measurements [30]. Both these studies were undertaken over a shorter period of time than the present study, and they involved MVC measurements, which are inherently impossible in animals [31].

Compartmentalisation of large muscles has been described for muscles acting on a number of joints (such as the longissimus dorsi muscle of the horse [32]) as well as for muscles acting on a single joint with a single point of insertion (such as the human deltoid muscle). In the human deltoid muscle an even more detailed study showed that anatomical and functional segments exist in this muscle, with seven anatomical segments identified based on the intramuscular tendons and at least two electrode positions (posterior and middle deltoid) with different activities of sEMG have been described [33].

The equine gluteus medius muscle which was examined in the present study has previously been investigated for signs of compartmentalisation with differences in fibre types of muscle fibres depending on the depth of the muscle [14] However, functional compartmentalisation of the equine gluteus has not been investigated using EMG. In the equine longissimus muscle activities along the thoracolumbar spine were found to peak at different time points during the motion cycles [11, 32] associated with the need for spinal stabilisation at these locations. This muscle has a clearly segmented structure, and each segment is innervated by the respective spinal nerve. Ritruechai et al. [33] demonstrated based on computed tomography scans the complex architecture of the equine longissimus dorsi muscle. Its different regions thus perform different mechanical functions; and the recruitment of different compartments within this muscle depends on the mechanics of the movement [34]. Such a clear differentiation was not found for the equine gluteus medius between the three EMG locations used in the present study. However, the equine gluteus medius muscle showed subtle differences in timing and extent of muscle activity measured by sEMG at the three electrode positions in the present; and, furthermore, these differences were quite variable for the horses measured. Such small differences may result in slightly different force vectors within the same muscle. They may allow muscles to fulfil a variety of functions while reducing the risk of damage to the muscle, its tendon or its origin/insertion. Varying muscle fibre use is also expected to allow more efficient blood flow to and from the muscle. In many muscles, such a functional differentiation is represented by different heads, bi- or multi-pennation, or an inner fascial layer separating muscle volumes; the function of the different heads of large muscles such as the equine triceps brachii and biceps brachii [35] has been documented. However, in the present study, no significant differences between patterns and between magnitudes of muscle activities of GM at the three electrode positions could be identified despite the large muscle volume investigated, and the first hypothesis is therefore rejected.

The three dimensional architecture and electrical activity of a thick muscle is not documented in its full complexity using sEMG. The main use of a muscle is reflected in its fibre type distribution, which is closely related to the lengths and velocities of muscle fibres, and the force that can be generated by it [2]. Preceding studies have shown that regional morphological variations of the longissimus dorsi muscle exist [31, 36] as the fascicles closer to the vertebral bodies experience smaller strains and strain rates than the more dorsal and lateral fascicles. In the GM, similar differences in fibre type distribution were found [14], and in the horses used for the present study, superficial muscle biopsies of GM were taken. These showed a relatively even distribution of about 30% each of Type 1, Type 2a and Type 2X fibres [16]; this result is similar in its Type1 versus Type 2 fibre distribution to a previous study [37], where the higher use of GM at the higher speeds was interpreted as a reflection on the relatively larger number of type IIB fibres (subsumed in the Type 2X fibres) in the periphery of those muscles. The connection of the muscle fibres of the deeper layers of thick muscles may be similarly related to intramuscular EMG readings of these layers, this is an option for future studies.

Over the last two decades GM was the equine limb muscle most commonly investigated using sEMG; however there is still no consensus over the most suitable electrode position on this muscle. In humans, the SENIAM project (Surface Electromyography for the Non-Invasive Assessment of Muscles) was established to provide such guidelines for sensor placement and signal processing [36] but in animals the location of the electrode positions is not yet standardized which is detrimental for the comparison of results [31].

The placement of electrodes over innervation zones is known to reduce the signal amplitude [38] this should therefore be avoided [39], however for the equine gluteus no such information is as yet available. Surface array methods have been used in humans to identify innervation zones, this may also be possible in animals, and should become available over the next decade.

Descriptions of the location for the placement of gluteus medius electrodes have not always been well specified; descriptions include the approximate middle of the muscular belly [40], over the assumed midpoint of the muscle belly [5] or the midway along the lengths of the muscle [20]. In the current study the electrodes were positioned in three closely adjacent locations, around the midpoint identified by palpation of the palpable bony prominences, with the aim of making at least one of the electrode positions comparable to the electrode positions used in the studies discussed above. This was achieved, as palpating the anatomical descriptions in the other studies [5, 20, 40] lead to a location very close to GM2 electrode.

The present study was subject to several methodological considerations.

The number of motion cycles available for analysis was different for walk and trot, as the data collection time for each trial was kept the same. On each data collection day, 18 motion cycles of walk and 39 motion cycles of trot were obtained. An alternative approach might have been to double the data collection time for the walk, resulting in roughly similar numbers. Such an approach might have detected more significant differences between walk and trot.

In most textbooks, the placement of sEMG electrodes over scars is discouraged [41]. However, in the present study one of the electrodes was positioned over the eight week old muscle biopsy scar on DCD3. Interestingly the results were not different from the results obtained from the other electrode at the same day, but there were differences detected in the pattern of activity (MML) when comparing with same position during preceding measurements. This was somewhat surprising, and may have been associated with the biopsy scar. It is possible, that the magnitude of the muscle activity is unaffected as the volume of muscle fibres sampled below the electrode is larger than the 1cm^3^ removed. On the other hand, the data processing chosen for the present study (normalisation to the maximum value) may have reduced the relevance of these fewer muscle fibres samples.

The present study was carried out with very little data processing, consisting of resampling, DC offset removal, rectification, and a low pass filter; this was followed by normalisation to the maximum value. Other studies reporting gluteal muscle activity in dogs during locomotion focussing on the description of the muscle function have applied different data processing steps, including rectification, generation of an EMG sample with 120 bins, and normalization of the EMG amplitude to the control trials [28].

Additionally, a high pass filter is used in many studies for sEMG data processing; however, this was not done in the present study based on the fact that the high pass filter employed may, in some instances reduce, or even remove pertinent information contained in the signal [42]. Also, no clear indications for the need of a high pass filter (such as wavering baselines) were noted in the EMG signals.

## Conclusions

The GM does not show any indication of segmentation when comparing three sEMG electrode positions during walk and trot on the treadmill; this might be due to a lack of segmentation per se (supported by anatomy) or due to a lack of need for segmented use for these very basic main tasks of the muscle. A scar of muscle biopsy (with about 1 cm^3^ muscle tissue removed) did not create measurable differences in magnitude of muscle activity, but differences found in pattern of activity may be associated with the scar. The reliability of the sEMG measurements over several weeks was fair to good, an indication for the robustness of the methodology.

## Abbreviations

@T: at trot
@W: at walk
CI: 95% confidence interval
DCD: Data collection day
GM: Gluteus medius muscle
GM1: Medial to the middle of the gluteus medius muscle
GM2: Central on the gluteus medius muscle
GM3: Lateral to the middle of the gluteus medius muscle
GYS1: Glycogen synthase mutation
IQR: Interquartile range
L: Left
MML: Mean mode locations
MMV: Mean mode value
R: Right
S.D.: Standard deviation
sEMG: Surface electromyography
St: Stance phase
Sw: Swing phase
